# Validating Type 2 Diabetes Diagnoses Across the Madrid Hospital Network: Evidence for Secondary Use of the CMBD in the European Health Data Space

**DOI:** 10.3390/jcm15197717

**Published:** 2026-10-07

**Authors:** Rafael Gómez-Coronado-Martín, Ana Chacón-García, Ana López-de-Andrés, Miguel A. Salinero-Fort, Daniala L. Weir, Carmen de Burgos-Lunar

**Affiliations:** 1 Servicio de Admisión y Documentación Clínica, Hospital Clínico San Carlos, Instituto de Investigación Sanitaria del Hospital Clínico San Carlos (IdISSC), 28040 Madrid, Spain; rafael.gomezcoronado@salud.madrid.org (R.G.-C.-M.); ana.chacon@salud.madrid.org (A.C.-G.); 2 Department of Public Health and Maternal & Child Health, Universidad Complutense de Madrid, Instituto de Investigación Sanitaria del Hospital Clínico San Carlos (IdISSC), 28040 Madrid, Spain; anailo04@ucm.es (A.L.-d.-A.); carmen.deburgos@salud.madrid.org (C.d.B.-L.); 3 Division of Pharmacoepidemiology & Clinical Pharmacology, Utrecht Institute for Pharmaceutical Sciences (UIPS), Utrecht University, 3584 CS Utrecht, The Netherlands; d.l.weir@uu.nl; 4 Network for Research on Chronicity, Primary Care, and Health Promotion (RICAPPS), Carlos III Health Institute, 28029 Madrid, Spain; 5 Research Network on Health Services and Chronic Diseases (REDISSEC), Carlos III Health Institute, 28029 Madrid, Spain; 6 Frailty, Multimorbidity Patterns and Mortality in the Elderly Population Residing in the Community Group— Hospital La Paz Institute for Health Research IdiPAZ, 28046 Madrid, Spain; 7 Community and Home Care Unit, Faculty of Biomedical and Health Sciences, Alfonso X El Sabio University, Villanueva de la Cañada, 28691 Madrid, Spain; 8 Servicio de Medicina Preventiva, Hospital Clínico San Carlos, Instituto de Investigación Sanitaria del Hospital Clínico San Carlos (IdISSC), 28040 Madrid, Spain

**Keywords:** CMBD, diagnostic accuracy, European Health Data Space, hospital discharge data, type 2 diabetes mellitus, validation study

## Abstract

**Background/Objectives:** Hospital discharge data are increasingly reused for research and within health data spaces, but their value depends on accurate diagnostic coding. The validity of type 2 diabetes mellitus (T2DM) coding in the Spanish hospital discharge database (CMBD) had not been assessed across a whole regional network. We aimed to quantify the precision and completeness of E11 coding across the public hospital network of the Region of Madrid. **Methods:** We conducted a retrospective validation study in the public hospitals of the Region of Madrid (2016–2025). In each of four hospital complexity groups, we selected admissions with and without an ICD-10-ES E11 code, matched by age and sex (787 and 781 analysed). The reference standard was the American Diabetes Association criteria, checked in health records. Predictive values were estimated directly; sensitivity, specificity and prevalence were reconstructed for the whole population, adjusting for sex and age. **Results:** Of 4,707,924 discharges, 918,797 (19.5%) had a T2DM code. The positive predictive value was 94.2% (95% CI: 92.1–96.0), the negative predictive value 94.0% (91.3–96.3), specificity 98.5% (98.0–99.0) and sensitivity 79.3% (72.4–86.2), a lower bound. The estimated prevalence of T2DM among discharges was 23.2%, against 19.5% coded; about 226,000 admissions with T2DM had no code. Only the positive predictive value differed between hospital groups (*p* = 0.006). **Conclusions:** The E11 code is highly precise but incomplete: it is reliable for building cohorts, whereas counts based on it underestimate the number of cases by about one in five.

## 1. Introduction

Routinely collected healthcare data have become a principal source of evidence on interventions, disease burden and health system performance [1,2,3], driven by information systems that make large-scale data capture and linkage feasible [1,4,5] and by the recognition that randomised trials cannot answer every question arising in routine practice [1,6]. The Spanish National Health Data Space [7] and the European Health Data Space (EHDS), established by Regulation (EU) 2025/327 [8], are being built on the premise that such records can be pooled, linked and reused across institutions and borders through infrastructures such as HealthData@EU [9]. That premise rests on the accuracy of the diagnostic codes that those records carry.

In Spain, the principal such record is the Minimum Basic Data Set on Hospital Discharges (Conjunto Mínimo Básico de Datos, CMBD), which has been mandatory for all National Health System hospitals since 1992 and extended to private centres in 2005 [10]. It covers every hospital discharge and records demographic and admission data, primary and secondary diagnoses coded in ICD-10, procedures and discharge destination, translated into codes from the discharge summary by staff trained specifically in clinical coding. It is already the most widely used administrative data source in Spanish health services research [11] and the natural Spanish contribution to a federated health data space.

The CMBD was, however, designed for administrative and financial purposes rather than for research [2,3,12,13,14]. The discharge summary may omit conditions peripheral to the reason for admission, and it is coded by staff who did not see the patient, so a condition may be coded when absent, or be present and never coded. Accuracy also varies widely by condition. Early studies of the Uniform Hospital Discharge Data Set, the U.S. precursor of the CMBD, reported error rates above 30% for the principal diagnosis [14,15] and later studies rates of approximately 20% [16,17], ranging from approximately 2% for pneumonia to 45% for acute myocardial infarction [18,19]. Spanish validations for cerebrovascular disease, venous thromboembolic disease, influenza, colorectal cancer and medication-related adverse events [20,21,22,23,24] and, more recently, for type 1 diabetes in a tertiary hospital of the network studied here [25] show the same dependence on the condition. For diabetes, a systematic review found consistently high specificity but sensitivity ranging from 59% to 92% across hospital discharge datasets [26], a spread too wide for estimates obtained in one setting to be assumed to hold in another.

Validity in this context comprises two quantities that answer different questions. A cohort study needs the positive predictive value, the proportion of coded admissions that genuinely have the condition; an estimate of disease burden or of a population denominator needs the sensitivity, the proportion of admissions with the condition that carry the code. Coding can be precise and incomplete at the same time, so a validation reporting only one of them leaves the other unknown. Which of the two a study can estimate directly depends on how its sample was drawn: when admissions are sampled on the result of the code rather than on disease status, the predictive values are estimated directly, whereas sensitivity and specificity must be reconstructed from the sampling fractions and the size of the corresponding population strata [27].

Type 2 diabetes mellitus (T2DM) is an obvious priority for such an exercise. It is among the most prevalent chronic conditions in hospitalised adults and is strongly associated with cardiovascular complications, repeated hospitalisation, premature mortality and a substantial economic burden on health systems [28,29,30]; an estimated 589 million adults were living with diabetes worldwide in 2024, more than 90% of them with T2DM [31]. It is also recorded in discharge data mostly as a comorbidity rather than as the reason for admission, precisely the position in which coding is most vulnerable to omission.

The validity of T2DM coding in the CMBD has nevertheless not been established: the available Spanish validations are single-centre or concern other conditions, and none have examined an entire regional hospital network. That scope matters because hospitals differ in case mix and in the organisation of their coding teams, and a health data space requires estimates anchored to the source population rather than to a single centre. We therefore set out to validate T2DM diagnoses recorded in the CMBD across the entire public hospital network of the Region of Madrid over a ten-year period against American Diabetes Association diagnostic criteria verified in the electronic health record to quantify both the precision and the completeness of E11 coding, overall and across hospital complexity groups, at the level of the source population.

## 2. Materials and Methods

### 2.1. Study Design and Reporting

This was a retrospective, cross-sectional study of the diagnostic accuracy of T2DM coding in the CMBD. The index test was the presence of ICD-10-ES code E11 in the hospital discharge record; the reference standard was fulfilment of the American Diabetes Association (ADA) diagnostic criteria for T2DM, verified by review of the electronic health record (EHR) [32]. The study is reported in accordance with the STARD 2015 guidelines for studies of diagnostic accuracy [33].

### 2.2. Setting

The study was conducted across the public hospital network of the Madrid regional health service (Servicio Madrileño de Salud, SERMAS), which provides universal coverage to the population of the Region of Madrid (more than 6.7 million inhabitants). The network comprises 35 public hospitals, classified by the regional health authority into four groups according to size, complexity and case mix, ranging from large tertiary teaching hospitals (Group 1) to small, medium-stay and specialised centres (Group 4). All adult discharges recorded in the CMBD across these centres during the study period were eligible, allowing accuracy to be assessed both overall and within each complexity group. Two centres not assigned to any complexity group (5715 discharges, 557 of them coded) were not sampled and are excluded from the population-level estimates.

### 2.3. Population and Unit of Analysis

The unit of analysis was the hospital admission (discharge episode). The study population comprised adult admissions recorded in the CMBD between 2016 and 2025, and the inclusion criterion was age 18 years or over at admission.

Selected admissions were excluded if they met either of the following criteria: (a) absence in the EHR of any laboratory determination permitting the ADA criteria to be applied, namely, plasma glucose (fasting or random), glycated haemoglobin (A1C) or an oral glucose tolerance test; or (b) absence of a Regional Personal Identification Code (Código de Identificación Personal Autonómico, CIPA), the unique patient identifier that gives access to the EHR. No admission was excluded under criterion (a). The CIPA is missing mainly in admissions of patients not registered in the SERMAS system (residents of other autonomous regions, uninsured foreign patients or patients treated privately within the public system) and in episodes without complete administrative registration.

### 2.4. Data Sources and Review Window

The CMBD identified eligible admissions and provided the index test. The reference standard was applied to individual-level data from patients’ EHRs, including hospital, primary care, and outpatient records. Reviewers examined information from the inception of the electronic record up to the discharge date of the index admission; information generated after discharge was not consulted. Information preceding the admission was included because T2DM is a chronic condition and the discharge code is intended to capture prevalent disease.

### 2.5. Sampling

Two samples of admissions were drawn, both selected on the result of the index test and both stratified by hospital complexity group.

Sample 1 comprised admissions with code E11 recorded in any of the 20 diagnostic positions of the CMBD, including all E11 subcodes, selected by simple random sampling within each complexity group. The subcodes observed in the analysed sample are listed in Supplementary Table S1. Codes for the other diabetes categories (E08, secondary; E09, drug-induced; E10, type 1; E13, other specified) and for gestational diabetes (O24) were not used for case selection. Under the official Spanish coding guidelines, diabetes of unspecified type is assigned to code E11.

Sample 2 was drawn from the same hospital population and time frame and comprised admissions without code E11 in any diagnostic position. No further exclusion was applied: admissions carrying a code for another diabetes category were eligible as controls, so misclassification between diabetes types remained observable. Because the probability of T2DM is strongly associated with age and differs by sex, control admissions were selected by exact 1:1 matching to the sampled cases on sex and age in years within the same complexity group. Matched-pair identifiers were not provided, and all analyses were conducted at the group level. The complete sampling and matching specifications, the achieved balance and the query defining the study universe and the assignment of hospitals to complexity groups are given in the Supplementary Materials, and the age distributions of the two samples are compared in Supplementary Figures S1 and S2.

**Figure 1:**
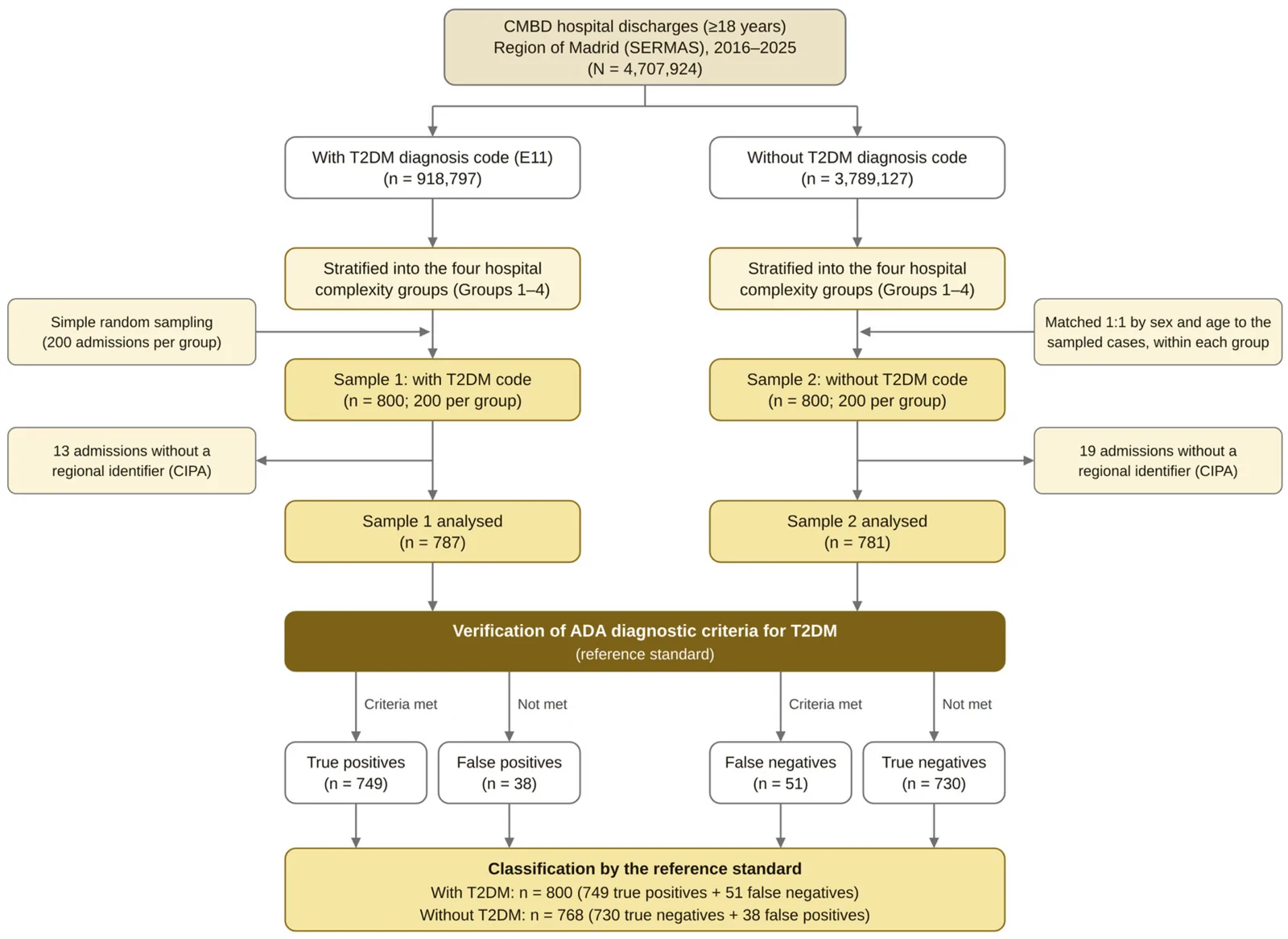
Flowchart of the study design and validation process of type 2 diabetes mellitus (T2DM) diagnoses in the Spanish National Hospital Discharge Database (CMBD) of the Madrid Regional Health Service (SERMAS), 2016–2025.

Assuming an expected proportion of concordant admissions of 98%, a 95% confidence level and an absolute precision of 2%, 189 admissions per stratum were needed; this was raised to 200 to allow for an estimated 5% loss from absent CIPA and multiplied by the four complexity groups, giving 800 admissions per sample and 1600 in total. Of these, 32 were excluded for the absence of a CIPA (13 in Sample 1 and 19 in Sample 2) and none for the absence of the required laboratory information, leaving 787 and 781 admissions analysed, respectively, and 1568 in total. The flow of admissions through the study is shown in Figure 1.

### 2.6. Reference Standard and Adjudication

The reference standard was fulfilment of the ADA diagnostic criteria for T2DM [32], applied to the laboratory results available in the EHR up to the index discharge. In nonpregnant adults, diabetes was defined by any of the following: A1C of 6.5% or more (48 mmol/mol or more), measured by an NGSP-certified method standardised to the DCCT assay; fasting plasma glucose of 126 mg/dL or more (7.0 mmol/L or more) after at least 8 h without caloric intake; 2-h plasma glucose of 200 mg/dL or more (11.1 mmol/L or more) during an oral glucose tolerance test performed according to WHO recommendations with a 75 g anhydrous glucose load; or, in a patient with classic symptoms of hyperglycaemia or hyperglycaemic crisis, a random plasma glucose of 200 mg/dL or more (11.1 mmol/L or more). In the absence of unequivocal hyperglycaemia, diagnosis required two abnormal results, either from two different tests obtained at the same time or from the same test at two different time points. The criteria are those of the 2025 Standards of Care; the diagnostic thresholds for T2DM have not changed since the beginning of the study period in 2016 [34].

Because qualifying values from any point in the recorded history were accepted, adequate glycaemic control at the time of the index admission did not preclude a positive classification. Conversely, a documented clinical diagnosis of T2DM or a prescription of glucose-lowering treatment was recorded during the review but was not by itself sufficient for a positive classification. The reference standard is therefore conservative with respect to specificity and positive predictive value, and the admissions affected by this rule are examined case by case in Supplementary Tables S5 and S6.

Each admission was assessed independently by two reviewers experienced in the management of EHRs. Both were blinded to the CMBD coding status of the admission, that is, to the presence or absence of code E11 and to the sample to which it belonged. Discrepancies between reviewers were resolved by a third reviewer.

### 2.7. Statistical Analysis

All analyses were performed in Python (version 3.12; NumPy 2.4.4, SciPy 1.17.1, pandas 3.0.2 and Matplotlib 3.10.8). Quantitative variables are summarised as the mean and standard deviation and, for skewed distributions, as the median and interquartile range; normality was assessed by inspection of histograms and quantile–quantile plots complemented by the Shapiro–Wilk test (Supplementary Table S8 and Figures S4 and S5). Categorical variables are summarised as absolute and relative frequencies, *n* (%). The representativeness of the validation sample with respect to the source population of E11-coded discharges was assessed using absolute standardised mean differences (SMDs), with values below 0.10 being taken as indicative of negligible differences [35]. Agreement between the CMBD code and the reference standard and agreement between the two reviewers were quantified using Cohen’s kappa with 95% confidence intervals (CIs); because kappa depends on the marginal distributions, which are fixed by the 1:1 design, it is reported as a property of the validation sample, and agreement between the code and the reference standard was additionally computed from the population classification table reconstructed as described below.

Because both samples were selected on the result of the index test (test-result-based sampling) [27], each is a probability sample of its own stratum of the source population. The predictive values were therefore estimated directly, as the proportion of reference-positive admissions in the coded sample and of reference-negative admissions in the non-coded sample, computed within each complexity group and weighted by the share of each group in the corresponding population stratum. Sensitivity and specificity cannot be obtained from the pooled 2 × 2 table because the two samples were drawn with different sampling fractions (787 of 918,240 coded and 781 of 3,783,969 non-coded discharges), an over-representation of coded admissions by a factor of approximately four, so that false negatives are correspondingly under-represented (Supplementary Table S2).

Sensitivity, specificity and the prevalence of T2DM among discharges were therefore obtained by reconstructing the population classification table, in line with current methodological guidance [36] and with comparable validation studies [37]. Within each complexity group h, with N+(h) coded and N-(h) non-coded discharges in the source population, the four cells were computed as TP(h) = N+(h) × p(D+|T+,h), FP(h) = N+(h) × [1 − p(D+|T+,h)], FN(h) = N-(h) × p(D+|T-,h) and TN(h) = N-(h) × [1 − p(D+|T-,h)], where p denotes the proportions observed in the corresponding sample; the accuracy measures were then computed from the summed table. The four complexity groups comprised 409,601, 241,589, 188,533 and 78,517 coded discharges and 1,873,275, 889,538, 715,992 and 305,164 non-coded discharges, respectively (Supplementary Table S4).

Because the control sample was individually matched to the coded sample on age and sex, it reproduces the age and sex structure of coded admissions rather than that of all non-coded discharges, which are younger and more often women. This distortion was corrected by post-stratification: the proportion of reference-positive admissions in the non-coded sample was estimated within ten cells defined by sex and five age bands (<60, 60–69, 70–79, 80–89 and 90 years or over) and weighted to the corresponding cells of the population of non-coded discharges. Because the regional data provider supplied the age and the sex distribution of discharges separately rather than jointly, the joint distribution was obtained from the two population margins by iterative proportional fitting (raking), seeded with the age–sex association observed in the control sample; the ten cells comprised between 27 and 147 controls. As a robustness check, the joint distribution was alternatively obtained by assuming independence of age and sex. Because this proportion did not differ significantly across hospital complexity groups, the post-stratified value was applied to the group-specific denominators of the reconstruction. The overall sensitivity, negative predictive value and prevalence reported here incorporate this correction; the group-specific estimates do not and are therefore slightly conservative for sensitivity and prevalence. Residual differences within cells cannot be excluded, so the reported sensitivity remains a lower bound and the prevalence an upper bound, whereas the positive predictive value is unaffected and the specificity essentially so. The Supplementary Materials set out the reconstruction in full, and Supplementary Table S3 compares the alternative weighting schemes.

Confidence intervals for the group-specific predictive values, which are simple sample proportions, were computed by the Wilson score method; those for the overall predictive values, which weight the groups, and for all reconstructed quantities, by parametric bootstrap (200,000 replicates, percentile method), resampling the classification counts of each sample and group from their binomial distributions; for the post-stratified estimates, the count of reference-positive admissions within each age–sex cell of the non-coded sample was likewise drawn from its binomial distribution, the population weights being treated as fixed. The population-level kappa was recomputed in each replicate. Two internal consistency checks were applied: the predictive values derived by Bayes’ theorem from the reconstructed sensitivity and specificity at the reconstructed prevalence must reproduce the directly estimated predictive values, and the Rogan–Gladen estimator [38] applied to the observed prevalence of coded discharges must return the same reconstructed prevalence.

Homogeneity across hospital complexity groups was assessed on the two proportions the design estimates directly. The proportion of coded admissions meeting the reference standard and the proportion of non-coded admissions not meeting it were each compared across the four groups by the chi-squared test on the corresponding classification counts, without continuity correction, with Fisher’s exact two-sided test where any expected frequency was below 5; the same tests were applied across sex and age strata. Homogeneity of the reconstructed sensitivity and specificity was assessed by an inverse-variance-weighted heterogeneity statistic on the logit scale with bootstrap variances, referred to a bootstrap null distribution rather than to the chi-squared approximation, which is unreliable at proportions close to one with approximately 200 admissions per group.

Three sensitivity analyses were performed: retaining a single admission per patient to verify that the small number of patients contributing two admissions did not influence the estimates; reclassifying as reference-positive those admissions with a documented diagnosis or glucose-lowering treatment but no verifiable qualifying laboratory value to bound the effect of the conservative reference standard (Supplementary Table S6); and estimating accuracy by year of discharge and by five-year period to examine temporal stability (Supplementary Table S7). In addition, the predictive values were computed across a range of plausible prevalences so that the estimates can be transported to populations other than the one studied (Supplementary Figure S3).

All estimates are reported with 95% CIs, and the significance level was set at 0.05. The population-level estimates are reported as the primary results; proportions observed within the validation sample are reported as descriptive of that sample.

## 3. Results

### 3.1. Source Population and Validation Sample

Between 2016 and 2025, 4,707,924 adult discharges were recorded in the CMBD of the public hospital network of the Region of Madrid, of which 918,797 (19.5%) carried at least one diagnosis coded as T2DM. Of the 1600 admissions selected, 32 were excluded for the absence of a CIPA (13 from Sample 1 and 19 from Sample 2) and none for the absence of the laboratory information required to apply the reference standard, leaving 787 coded and 781 non-coded admissions analysed. The complete flow is shown in Figure 1.

The 1568 analysed admissions corresponded to 1566 unique patients; two patients contributed two admissions each, in no case to both samples. A sensitivity analysis retaining one admission per patient yielded virtually identical classification proportions.

The characteristics of the source population of T2DM-coded discharges and of the corresponding validation sample are presented in Table 1. The sample was similar to the source population in demographic and clinical characteristics, with all absolute standardised mean differences below 0.10. By design, however, it was not proportional to the hospital-complexity-group structure of the source population: approximately 200 admissions were sampled from each group, so Group 1 accounted for 44.6% of T2DM-coded discharges in the source population but 24.8% of the validation sample, and Group 4 accounted for 8.5% and 24.4%, respectively (absolute standardised mean differences 0.425 and 0.437).

In the source population, T2DM-coded discharges were predominantly those of older adults (mean age 75.3 years), with a lower proportion of women (43.0%) and a high comorbidity burden, including renal disease (25.0%), heart failure (23.9%) and malignant neoplasms (15.3%). The annual distribution of T2DM-coded discharges was stable across the study period. Among the 781 analysed control admissions, nine carried a code for another diabetes category (five E09, three E10 and one E13), so misclassification between diabetes types remained observable in the study.

### 3.2. Classification of the Validation Sample and Interobserver Agreement

Of the 787 coded admissions, 749 met the reference standard, and 38 did not; of the 781 non-coded admissions, 730 did not meet it, and 51 did. The classification counts by sex, age group and hospital complexity group, with the corresponding proportions, are given in Table 2.

**Table 2:** Classification of the validation sample against the reference standard by sex, age group and hospital complexity group.

Stratum	TP	FP	FN	TN	Coded Admissions Meeting the Reference Standard, % (95% CI)	Non-Coded Admissions Not Meeting It, % (95% CI)	Kappa (95% CI)
Overall	749	38	51	730	95.17 (93.44–96.46)	93.47 (91.52–95.00)	0.886 (0.864–0.909)
Female	328	16	16	326	95.35 (92.58–97.12)	95.32 (92.54–97.10)	0.907 (0.875–0.938)
Male	421	22	35	404	95.03 (92.60–96.70)	92.03 (89.11–94.21)	0.871 (0.838–0.903)
<70 years	197	14	13	196	93.36 (89.17–96.01)	93.78 (89.65–96.33)	0.871 (0.825–0.918)
70–<80 years	227	6	16	211	97.42 (94.50–98.81)	92.95 (88.86–95.62)	0.904 (0.865–0.943)
80 years or over	325	18	22	323	94.75 (91.86–96.66)	93.62 (90.53–95.75)	0.884 (0.849–0.919)
Group 1	177	18	16	179	90.77 (85.88–94.08)	91.79 (87.09–94.89)	0.826 (0.770–0.882)
Group 2	193	7	14	184	96.50 (92.95–98.29)	92.93 (88.48–95.74)	0.894 (0.851–0.938)
Group 3	196	4	14	183	98.00 (94.97–99.22)	92.89 (88.43–95.72)	0.909 (0.868–0.950)
Group 4	183	9	7	184	95.31 (91.33–97.51)	96.34 (92.63–98.21)	0.916 (0.876–0.957)

Proportions and kappa are properties of the validation sample under its 1:1 design. Because each sample was drawn on the result of the index test, the two proportions estimate the positive and negative predictive value within the corresponding stratum; they are carried to the population level in Table 3. Homogeneity across sex strata: *p* = 0.838 (coded arm) and *p* = 0.064 (non-coded arm); across age strata: *p* = 0.122 and *p* = 0.930; chi-squared test without continuity correction. Wilson score confidence intervals. TP: true positive; FP: false positive; FN: false negative; TN: true negative; CI: confidence interval.

**Table 3:** Accuracy of E11 coding and estimated prevalence of T2DM at the population level, overall and by hospital complexity group.

	Sensitivity % (95% CI)	Specificity % (95% CI)	PPV % (95% CI)	NPV % (95% CI)	Estimated Prevalence of T2DM % (95% CI)
Overall (primary)	79.3 (72.4–86.2)	98.5 (98.0–99.0)	94.2 (92.1–96.0)	94.0 (91.3–96.3)	23.2 (21.3–25.4)
Overall, before post-stratification	75.6 (70.3–81.4)	98.5 (98.0–99.0)	94.2 (92.1–96.0)	92.6 (90.3–94.8)	24.3 (22.6–26.2)
Group 1	70.8 (61.7–81.3)	97.8 (96.9–98.7)	90.8 (85.9–94.1)	91.8 (87.1–94.9)	23.0 (19.9–26.4)
Group 2	78.8 (70.7–87.9)	99.0 (98.2–99.7)	96.5 (93.0–98.3)	92.9 (88.5–95.7)	26.2 (23.5–29.2)
Group 3	78.4 (70.3–87.8)	99.4 (98.9–99.9)	98.0 (95.0–99.2)	92.9 (88.4–95.7)	26.1 (23.3–29.1)
Group 4	87.0 (79.1–95.8)	98.8 (97.9–99.5)	95.3 (91.3–97.5)	96.3 (92.6–98.2)	22.4 (20.4–24.8)
p, homogeneity across groups	0.19	0.10	0.006	0.30	-

Predictive values are estimated directly within each group and weighted to the population structure in the overall rows; sensitivity, specificity and the prevalence are reconstructed from the population classification table (Methods). The primary row incorporates the sex and age post-stratification of the non-coded arm; the second row reports the overall estimates before that correction and is therefore the exact combination of the four group rows. Post-stratification does not affect the positive predictive value; 95% CIs by the Wilson score method for the group-specific predictive values and by parametric bootstrap (200,000 replicates) otherwise. *p*-values: chi-squared test for the predictive values; bootstrap-calibrated heterogeneity statistic on the logit scale for sensitivity and specificity, computed on the estimates before post-stratification. PPV: positive predictive value; NPV: negative predictive value; CI: confidence interval. T2DM: type 2 diabetes mellitus.

Agreement between the CMBD code and the reference standard within the validation sample was high, with a kappa coefficient of 0.886 (95% CI: 0.864–0.909) [40]. Agreement between the two independent reviewers was excellent: kappa 0.944 (95% CI: 0.927–0.960), with 97.2% of admissions classified identically. Because the marginal distributions of the validation sample are fixed by the 1:1 design, this kappa describes the sample rather than the source population; the corresponding value reconstructed at the population level is reported in Section 3.4.

### 3.3. Predictive Values of E11 Coding

Because each sample is a probability sample of its own stratum of the source population, the proportions in Table 2 estimate the predictive values directly. Weighted to the hospital-complexity-group structure of the source population, the positive predictive value was 94.2% (95% CI: 92.1–96.0); the negative predictive value, which additionally incorporates the post-stratification of the non-coded arm, was 94.0% (95% CI: 91.3–96.3) (Table 3). The positive predictive value is slightly lower than the pooled sample proportion of 95.2% because Group 1, which has the lowest value, accounts for the largest share of coded discharges.

The 38 coded admissions that did not meet the reference standard were reviewed case by case and fell into five patterns: hyperglycaemia in the context of corticosteroid therapy or acute illness (12), documented prediabetes or impaired fasting glucose (9), glucose-lowering treatment without verifiable qualifying laboratory values (7), latent autoimmune diabetes of adults or type 1 diabetes (4), and absence of laboratory values in the diabetic range (6) (Supplementary Table S5). Reclassifying as reference-positive the seven admissions in which a diagnosis could not be verified despite documented treatment raised the pooled sample positive predictive value from 95.2% to 96.1% and left the negative predictive value unchanged (Supplementary Table S6).

### 3.4. Accuracy and Prevalence of T2DM at the Population Level

Reconstructed to the source population, with the non-coded arm post-stratified by sex and age band, the sensitivity of E11 coding was 79.3% (95% CI: 72.4–86.2) and its specificity 98.5% (95% CI: 98.0–99.0). Both internal consistency checks held: the predictive values derived by Bayes’ theorem from these estimates at the reconstructed prevalence reproduced the directly estimated values, and the Rogan–Gladen estimator applied to the observed prevalence of coded discharges returned the same reconstructed prevalence.

The estimated prevalence of T2DM among adult discharges was 23.2% (95% CI: 21.3–25.4), against 19.5% of discharges carrying an E11 code. Applied to the source population, this corresponds to approximately 226,000 admissions over the ten-year period that met the diagnostic criteria for T2DM but carried no E11 code. Reconstructed at the population level, the overall agreement between the code and the reference standard was kappa 0.82 (95% CI: 0.77–0.88).

These overall estimates correct the age and sex distortion introduced by the matched control sample. The proportion of reference-positive admissions among non-coded discharges was 6.5% in the matched sample as drawn and 7.4% when that sample was weighted to the hospital-complexity-group structure of the source population, but 6.0% when it was post-stratified to the joint age and sex structure of non-coded discharges, and 5.8% under the alternative assumption that age and sex are independent (Supplementary Table S3). The post-stratified estimate raises the reconstructed sensitivity from 75.6% to 79.3% and lowers the estimated prevalence from 24.3% to 23.2%, leaving the positive predictive value unchanged and the specificity essentially so.

Because predictive values depend on the prevalence of the condition in the population to which the code is applied, Table 4 reports them across a range of prevalences derived from the reconstructed sensitivity and specificity so that the estimates can be transported to other settings. The range examined brackets the prevalence estimated here and the values reported in other hospitalised populations [41,42,43,44], and Supplementary Figure S3 shows the same relationship separately for each hospital complexity group.

### 3.5. Variation Across Hospitals and over Time

The proportion of E11-coded admissions meeting the reference standard differed across hospital complexity groups (*p* = 0.006), from 90.8% in Group 1 to 98.0% in Group 3; expressed the other way, 9.2% of coded admissions in Group 1 did not meet the reference standard, against 2.0% in Group 3. The proportion of non-coded admissions not meeting it did not differ (*p* = 0.30), nor did sensitivity (*p* = 0.19). Specificity varied over a narrow range (97.8% to 99.4%; *p* = 0.10) and was attenuated relative to the positive predictive value because it applies to the much larger reference-negative denominator (Table 3).

The estimated prevalence of T2DM among discharges was similar across the four groups (22.4% to 26.2%), so this gradient is a property of the coding rather than of the populations coded. Population-level estimates by group are also reported in Supplementary Table S4.

The precision was stable over the study period. The proportion of E11-coded admissions meeting the reference standard was essentially identical in the two halves of the study, 95.2% (95% CI: 92.6–97.0) in 2016–2020 and 95.1% (92.6–96.8) in 2021–2025 (*p* = 0.96). The proportion of non-coded admissions not meeting the reference standard rose from 92.0% (88.8–94.4) to 94.8% (92.2–96.6), a difference that did not reach statistical significance (*p* = 0.11) but is in the direction of fewer uncoded cases in recent years. Neither proportion varied significantly across the ten individual years (*p* = 0.86 and *p* = 0.36). Reconstructed to the discharges of each period without the sex and age post-stratification, which cannot be applied period by period, the positive predictive value was unchanged (94.3% and 94.0%), whereas sensitivity rose from 70.5% to 80.6%, a difference of 10.1 percentage points (95% CI: −1.0 to 20.7) that did not reach conventional statistical significance; these two figures are comparable with each other but not with the overall estimate (Supplementary Table S7).

## 4. Discussion

This region-wide validation shows that the E11 code in the CMBD is highly precise but incomplete and that these two properties differ markedly. When the code is present, it is almost always correct: 94 of every 100 coded admissions met the ADA diagnostic criteria on review of the electronic record, and fewer than two in every hundred admissions without T2DM carried the code. When the code is absent, however, its absence cannot be read as evidence that the condition is absent: approximately one in five admissions meeting the diagnostic criteria carried no E11 code, so that the prevalence of T2DM among adult discharges exceeds the prevalence of coded discharges by close to four percentage points. A validation reporting only one of these two properties would have given a misleading picture of the data source, and which of them matters depends entirely on the analysis the data are being used for.

The precision of the code is, if anything, understated by our estimates. The reference standard was strictly laboratory-based, and a documented clinical diagnosis or a prescription of glucose-lowering treatment was not by itself accepted as confirmation. Discordant admissions coded as T2DM are, by definition, false positives relative to the reference standard. However, the case-by-case review shows that they do not represent a homogeneous group of coding errors. Twenty-eight of the 38 corresponded to clinically recognisable situations in which a diagnosis of diabetes is plausible but the criteria were not formally met in the record—hyperglycaemia during corticosteroid therapy or acute illness, documented prediabetes, or treatment without verifiable qualifying values—and a further four corresponded to autoimmune diabetes, which is a misclassification of diabetes type rather than of diabetes itself. The direction of any residual error is therefore known: reclassifying the admissions in which treatment was documented but no laboratory value could be verified raises the positive predictive value rather than lowering it. A further contributor is structural: under the official Spanish coding guidelines, diabetes of unspecified type is assigned to code E11, so that an E11 code does not always represent a positive assertion of type 2 disease by the coder.

The under-coding we observed is the more consequential finding. Because residual age and sex differences within the post-stratification cells would, if anything, raise the reconstructed sensitivity further, the proportion of admissions with T2DM carrying no code is at most about one in five. A single-centre study at a U.S. university hospital found that between 5% and 15% of hospitalised patients with clinical or laboratory evidence of diabetes lacked a documented diagnosis in the discharge record [45]. Our upper bound is therefore of the same order and somewhat higher, although the health systems, coding classifications and case ascertainment differ. What it does not support is the reading that omission of chronic comorbidities has been eliminated from discharge coding in this setting.

Our estimates sit comfortably within the published literature. A systematic review of validated case definitions for diabetes in ICD-9- and ICD-10-coded data reported, for hospital discharge datasets, sensitivity ranging from 59% to 92%, specificity from 95.5% to 99% and positive predictive value from 62.5% to 96% [26]. The sensitivity estimated here falls within that range, the specificity within its upper portion, and the positive predictive value near its upper bound. In Canada, administrative definitions combining hospital and primary care diagnoses have achieved sensitivities of 92% to 95% for population surveillance [26,46], but that comparison is instructive rather than unfavourable: those definitions draw on outpatient records as well as hospital records, and a broader definition that captures nearly all known cases does so at the cost of admitting false positives—the authors of one such algorithm reported a positive predictive value of 77% in a community population [46]. Our results describe what a single hospital discharge code achieves on its own, and they suggest that the way to raise completeness in the CMBD is not to relax the coding of E11 but to link hospital discharge data to other sources.

The region-wide design allowed coding accuracy to be compared across hospitals of different complexity, which a single-centre study cannot do. The difference between groups was confined to the precision of the code, which was lowest in the largest tertiary hospitals; completeness did not differ, and the similar prevalence of T2DM across the four groups indicates that the gradient reflects the coding rather than the populations coded. Our data do not identify its cause; establishing whether it relates to case mix, coding workload or the organisation of coding teams would require information on the coding process itself, which this study did not collect.

These findings translate into concrete guidance for secondary use. For analyses that begin from a cohort of coded admissions—describing patients with T2DM, comparing outcomes between them, or evaluating care processes—the E11 code is a sound selection criterion, and the residual contamination is small and of known direction. For analyses that require a complete count—estimating the burden of T2DM among hospitalised adults, constructing denominators, or comparing rates between populations—the code alone will undercount cases by a margin large enough to matter, and the undercount is not uniform across hospitals. The distinction is not academic: the same validated code is fit for one purpose and requires correction for the other, and the correction factor is what this study provides. Predictive values, moreover, transport to other settings only through sensitivity, specificity and the prevalence of the target population, which is why we report them across a range of prevalences rather than as a single figure.

The high precision we observed is consistent with how discharge coding is organised in Spain. Diagnoses have been coded in a standardised manner using ICD-10-ES since 2016 by staff holding a nationally regulated vocational qualification, Técnico Superior en Documentación y Administración Sanitarias, a two-year programme that includes competencies in clinical documentation and health information management. This common training framework may help explain why, when a code is assigned, it is assigned accurately, and it answers the concerns raised in the 1990s about discharge summaries being interpreted and coded by staff without specific preparation [47]. It does not, however, address completeness, which depends on whether the condition reaches the discharge summary in the first place—a clinical documentation step that precedes coding and lies outside the coder’s control. Our results suggest that the professionalisation of coding has addressed the first problem more thoroughly than the second.

To our knowledge, this is the first validation of T2DM coding in the Spanish CMBD and the first in any Spanish region to cover an entire public hospital network. The only related precedent we identified is a study in Extremadura that assessed CMBD as a source for epidemiological surveillance of type 1 diabetes in children and adolescents and concluded that it was insufficient for monitoring incidence [48]; our findings for T2DM in adults are more favourable, although they point in the same direction regarding completeness. The principal strengths of this work are the direct validation approach, based on patient-level review of clinical records and laboratory results rather than on algorithmic or indirect approximation, the blinding of reviewers to coding status, and the anchoring of every estimate to the structure of the source population rather than to that of the validation sample.

Several limitations should be borne in mind. First, the two validation samples were selected on the result of the index test, which is what allows the predictive values to be estimated directly but requires sensitivity and specificity to be reconstructed; those two estimates therefore rest on the assumption that the proportions observed within each sample transport to the corresponding population stratum. Second, and relatedly, the control sample was individually matched to the coded sample on age and sex, so it reproduces the age and sex structure of coded admissions rather than that of all non-coded discharges; this was corrected by post-stratifying the non-coded arm to the population age and sex margins, but residual differences within cells cannot be excluded, so the reconstructed sensitivity remains a lower bound and the estimated prevalence an upper bound (Supplementary Table S3). Third, the reference standard was strictly laboratory-based and verified in the electronic records, so admissions whose criteria could not be confirmed from the available information were classified as reference-negative, which is conservative with respect to specificity and positive predictive value. Fourth, controls were drawn from hospital admissions, which may introduce spectrum bias, as hospitalised patients differ from the general population in comorbidity burden and in the likelihood of undergoing laboratory testing [49]. Fifth, private hospitals were not included, which limits generalisability to that setting, although publicly financed care accounts for approximately 71% of hospital discharges in the region [50]. Sixth, admissions without a regional patient identifier (CIPA), 2% of the sampled admissions and concentrated among patients outside the regional health system, could not be verified and were excluded, so the results do not describe that group. Finally, although the overall sample size allowed precise estimation, some subgroup estimates—particularly within individual complexity groups and the younger age strata—rest on smaller numbers and should be read accordingly.

Beyond confirming the validity of T2DM coding, this study is directly relevant to the secondary use of health data now being organised in Spain and Europe. The CMBD is being incorporated into the Spanish National Health Data Space [7], which will in turn be federated with the European Health Data Space created by Regulation (EU) 2025/327 [8] through infrastructures for the secure secondary use of health data such as HealthData@EU [9]. Validation of a single diagnosis in a single regional database does not by itself demonstrate that the CMBD is fit for large-scale federated analyses. Rather, the present study provides disease-specific evidence on the validity of the ICD-10 E11 code in a large regional hospital network and illustrates a methodology that could be applied to other diagnoses and settings. Further validation studies covering additional conditions, populations and databases would be required before broader conclusions regarding the suitability of the CMBD for federated analyses could be drawn.

### Future Perspectives

Three lines of work follow from these results. The first is to extend this validation approach to other highly prevalent chronic conditions coded in the CMBD, such as hypertension, and to build in periodic revalidation, since coding practices evolve with training, classification updates and changes in hospital information systems. The second is to test whether linkage between hospital discharge data and primary care or pharmacy records recovers the admissions that hospital coding misses, which the international evidence suggests is the most promising route to improving completeness [26,46]. The third is methodological: the individual review of clinical records remains the bottleneck in studies of this kind, and machine learning and large language models may allow it to be partially automated, making revalidation feasible at greater frequency and across more conditions [51].

## 5. Conclusions

T2DM diagnoses coded in the CMBD accurately but incompletely identify hospitalised adults with the condition. Across the public hospital network of the Region of Madrid, 94% of admissions carrying an E11 code met the American Diabetes Association diagnostic criteria on review of the electronic health record, and fewer than two in every hundred admissions without the condition carried the code. At the same time, approximately one in five admissions that met the criteria carried no code, so that the prevalence of T2DM among adult discharges exceeded the coded prevalence by close to four percentage points. Precision was somewhat lower in the largest tertiary hospitals, whereas completeness did not differ across hospital complexity groups.

These two properties support different uses. The E11 code is a sound criterion for assembling cohorts of hospitalised adults with T2DM and for analyses conducted within them, whereas estimates of disease burden, health care utilisation or population denominators require the undercount reported here to be considered. On that basis, the findings support the reuse of the CMBD for epidemiological research, clinical evaluation and health outcome analysis and provide the kind of disease-specific validation evidence that the Spanish National Health Data Space and the European Health Data Space (Regulation (EU) 2025/327) require of a contributing data source.

These conclusions apply to adult admissions in the public hospitals of the Region of Madrid for which linkable electronic health records are available. They do not extend to private hospitals, other regions of Spain, outpatient care, or every purpose for which the CMBD might be used, and periodic revalidation will be needed as coding practices evolve.

## Figures and Tables

**Table 1:** Representativeness of the T2DM validation sample compared with all CMBD discharges coded as T2DM during 2016–2025.

		Sample of Discharges with T2DM Diagnosis (*n* = 787)	Discharges with T2DM Diagnosis (*n* = 918,797)	|SMD|
Gender	*n* (% females)	344 (43.7)	394,857 (43.0)	0.015
Age (years)	Mean (SD)	76.2 (12.1)	75.3 (12.1)	0.077
	Median (IQR)	77.7 (68.9–85.7)	77 (68–85)	
<60	*n* (%)	83 (10.5)	101,252 (11.0)	0.015
60-<70		128 (16.3)	169,308 (18.4)	0.057
70-<80		233 (29.6)	269,743 (29.4)	0.005
80-<90		268 (34.1)	284,541 (31.0)	0.066
≥90		75 (9.5)	93,953 (10.2)	0.023
Length of stay	Mean (SD)	11 (79.1)	8.5 (40.8)	0.039
	Median (IQR)	6 (2–10)	5 (3–10)	
Year of hospital discharge	*n* (%)			
2016		69 (8.8)	79,961 (8.7)	0.002
2017		79 (10)	85,729 (9.3)	0.024
2018		73 (9.3)	88,856 (9.7)	0.013
2019		79 (10)	92,111 (10.0)	<0.001
2020		76 (9.7)	85,883 (9.3)	0.011
2021		84 (10.7)	87,298 (9.5)	0.039
2022		88 (11.2)	94,134 (10.2)	0.03
2023		82 (10.4)	97,964 (10.7)	0.008
2024		85 (10.8)	102,075 (11.1)	0.01
2025		72 (9.1)	104,786 (11.4)	0.074
Type 2 DM as main diagnosis	*n* (%)	21 (2.7)	23,672 (2.6)	0.006
Hospital Group	*n* (%)			
Group 1		195 (24.8)	409,601 (44.6)	0.425
Group 2		200 (25.4)	241,589 (26.3)	0.02
Group 3		200 (25.4)	188,533 (20.5)	0.117
Group 4		192 (24.4)	78,517 (8.5)	0.437
Dementia	*n* (%)	65 (8.3)	75,516 (8.2)	0.001
Malignant neoplasm	*n* (%)	96 (12.2)	140,975 (15.3)	0.091
Myocardial infarction	*n* (%)	63 (8.0)	90,872 (9.9)	0.066
Cerebrovascular disease	*n* (%)	94 (11.9)	102,672 (11.2)	0.024
Heart failure	*n* (%)	186 (23.6)	219,763 (23.9)	0.007
Renal disease	*n* (%)	209 (26.6)	229,569 (25.0)	0.036
Death	*n* (%)	50 (6.4)	65,301 (7.1)	0.03
Charlson Index	Mean (SD)	2.1 (2.1)	2.2 (2.2)	0.058
	Median (IQR)	2 (0–3)	2 (0–3)	

SD: standard deviation; IQR: interquartile range; SMD: standardised mean difference; T2DM: type 2 diabetes mellitus; DM: diabetes mellitus. The Charlson comorbidity index was computed from the ICD-10 codes recorded in any diagnostic position using the updated weights of Quan et al. [39]; under this weighting, uncomplicated diabetes scores 0 and diabetes with chronic complications scores 1.

**Table 4:** Predictive values of E11 coding as a function of the prevalence of T2DM (sensitivity analysis).

Prevalence of T2DM	PPV % (95% CI)	NPV % (95% CI)
5%	73.7 (67.0–80.8)	98.9 (98.5–99.3)
10%	85.6 (81.1–89.9)	97.7 (97.0–98.5)
15%	90.4 (87.2–93.4)	96.4 (95.3–97.6)
20%	93.0 (90.6–95.2)	95.0 (93.4–96.6)
23.2% ^a^	94.2 (92.1–96.0)	94.0 (92.2–95.9)
30%	95.8 (94.3–97.2)	91.7 (89.3–94.3)

Computed by Bayes’ theorem from the reconstructed population-level sensitivity and specificity; 95% CIs by parametric bootstrap. ^a^ Estimated prevalence of T2DM among discharges in the source population; at this prevalence, the derived point estimates reproduce the directly estimated predictive values of Table 3; their confidence intervals, propagated from the reconstructed sensitivity and specificity, need not coincide with those of Table 3. PPV: positive predictive value; NPV: negative predictive value; CI: confidence interval. T2DM: type 2 diabetes mellitus.

## Data Availability

The data underlying this study are derived from the Hospital Discharge Database (CMBD) of the Madrid Regional Health Service (SERMAS). These data are not publicly available due to legal and ethical restrictions, as they contain confidential patient information, and validation requires access to individual medical records.

## Supplementary Materials

The following supporting information can be downloaded at: https://www.mdpi.com/article/10.3390/jcm15197717/s1. Figure S1: Kernel density of age at discharge, Sample 1 (E11-coded) versus Sample 2 (non-coded); Figure S2: Quantile–quantile plot of age at discharge, Sample 1 versus Sample 2; Table S1: E11 codes observed in the 787 analysed case admissions; Table S2: Dependence of the pooled sample estimates on the ratio of control to case admissions; Table S3: Sensitivity of the population-level estimates to the weighting of the non-coded arm; Table S4: Diagnostic validity of T2DM coding in the CMBD by hospital complexity group; Figure S3: Positive predictive value of code E11 as a function of the prevalence of T2DM, by hospital complexity group; Table S5: Categories of the 38 false-positive admissions; Table S6: Positive predictive value under the observed classification and after reclassifying the category-4 admissions as true positives; Table S7: Classification of the validation sample by year of discharge and study period; Table S8: Distribution and normality of the continuous variables; Figure S4: Distribution of age in the analysed admissions; Figure S5: Distribution of length of stay in the analysed admissions; Table S9: STARD 2015 checklist.

## Abbreviations

ADA	American Diabetes Association
CMBD	Conjunto Mínimo Básico de Datos (Spanish National Hospital Discharge Database)
EHR	Electronic Health Record
HCSC	Hospital Clínico San Carlos
ICD-10	International Classification of Diseases, 10th Revision
NPV	Negative Predictive Value
PPV	Positive Predictive Value
T1DM	Type 1 Diabetes Mellitus
T2DM	Type 2 Diabetes Mellitus
