# Supplementary material for: Validating Type 2 Diabetes Diagnoses Across the Madrid Hospital Network: Evidence for Secondary Use of the CMBD in the European Health Data Space
Source: J Clin Med. 2026 Oct 7;15(19):7717. doi: 10.3390/jcm15197717 (PMC13666438; doi:10.3390/jcm15197717)
Supplement: Supplementary file 1 [file jcm-15-07717-s001.zip › jcm-4556741-supplementary.pdf]

Supplementary Materials

Selection of the validation samples

Sampling was performed by the regional data provider on the sampling frame of all eligible adult admissions recorded in the CMBD of the SERMAS public hospital network between 2016 and 2025. Within each of the four hospital complexity groups, 200 admissions with code E11 were selected by simple random sampling, and 200 admissions without code E11 were selected by exact 1:1 matching to the selected cases on sex and age in years. Matched-pair identifiers were not provided, and all analyses were conducted at the group level.

Balance was verified on the 1,600 selected admissions: 451 men and 349 women in each sample; mean age 76.18 (SD 12.13) years in cases and 76.15 (SD 12.14) years in controls (difference 0.03 years, 95% CI: −1.16 to 1.22; standardised mean difference <0.10); and the age-group distributions differed by at most two admissions per stratum.

The query below defines the study universe and the assignment of hospitals to the four complexity groups.

```
-- Study universe: adult discharge episodes in the CMBD, 2016–2025 (SQL Server)
SELECT
    d.centre_id,
    COALESCE(g.complexity_group, 0) AS complexity_group,  -- 0: centre not classified
    d.age,
    d.sex,
    DATEDIFF(DAY, d.admission_date, d.discharge_date) AS length_of_stay,
    CASE WHEN d.dx01 LIKE 'E11%' OR d.dx02 LIKE 'E11%' OR d.dx03 LIKE 'E11%'
           OR d.dx04 LIKE 'E11%' OR d.dx05 LIKE 'E11%' OR d.dx06 LIKE 'E11%'
           OR d.dx07 LIKE 'E11%' OR d.dx08 LIKE 'E11%' OR d.dx09 LIKE 'E11%'
           OR d.dx10 LIKE 'E11%' OR d.dx11 LIKE 'E11%' OR d.dx12 LIKE 'E11%'
           OR d.dx13 LIKE 'E11%' OR d.dx14 LIKE 'E11%' OR d.dx15 LIKE 'E11%'
           OR d.dx16 LIKE 'E11%' OR d.dx17 LIKE 'E11%' OR d.dx18 LIKE 'E11%'
           OR d.dx19 LIKE 'E11%' OR d.dx20 LIKE 'E11%'
         THEN 1 ELSE 0 END AS e11_coded     -- E11 in any of the 20 diagnostic positions
INTO study_universe
FROM cmbd_discharges AS d            -- annual CMBD tables 2016–2025 combined with UNION ALL
LEFT JOIN hospital_group AS g        -- regional classification of the 35 SERMAS hospitals
       ON g.centre_id = d.centre_id  -- Groups 1 to 4: 7, 8, 9 and 11 hospitals
WHERE d.age >= 18
  AND YEAR(d.discharge_date) BETWEEN 2016 AND 2025;
```

Figures S1 and S2 compare the age distributions of the analysed admissions (n = 787 and n = 781; t test, p = 0.957; Mann–Whitney test, p = 0.943; Kolmogorov–Smirnov test, p = 1.000). After the exclusion of the 32 admissions without a CIPA, the samples comprised 443 and 439 men and 344 and 342 women.

Figure S1. Kernel density of age at discharge, Sample 1 (E11-coded) versus Sample 2 (non-coded).

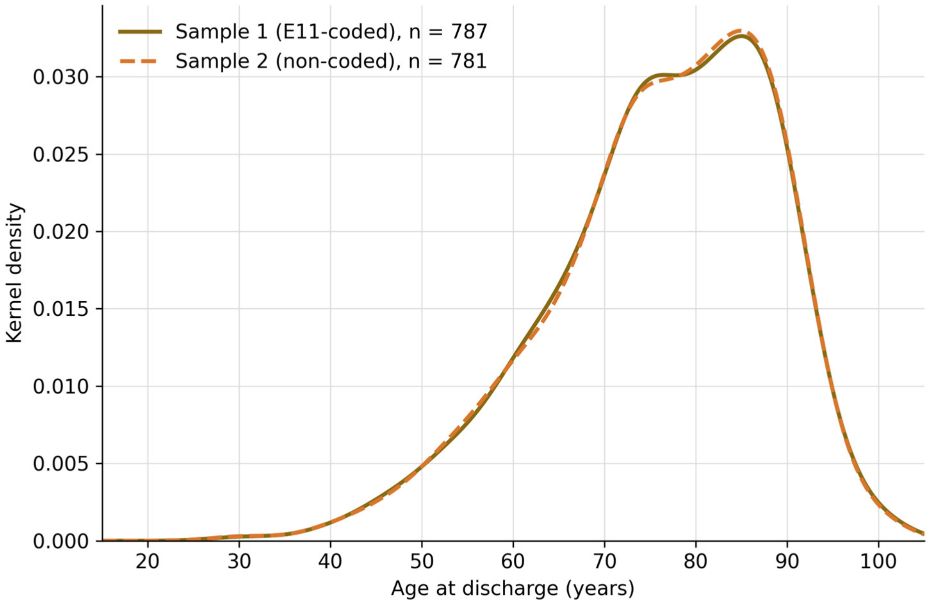

Figure S2. Quantile–quantile plot of age at discharge, Sample 1 versus Sample 2.

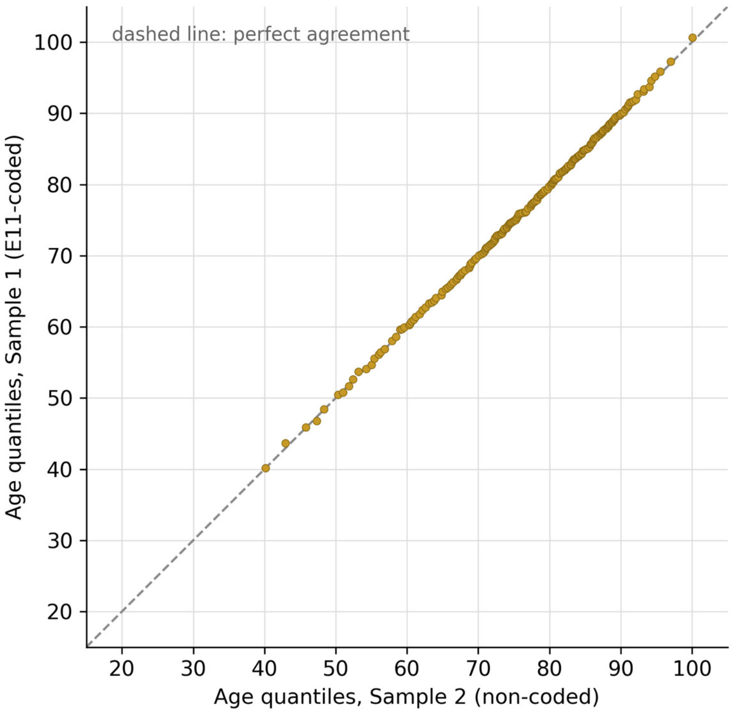

Table S1. E11 codes observed in the 787 analysed case admissions.

Code	Description	Case admissions, n
E11.00	T2DM with hyperosmolarity without nonketotic hyperglycaemic-hyperosmolar coma	3
E11.10	T2DM with ketoacidosis without coma	3
E11.21	T2DM with diabetic nephropathy	14
E11.22	T2DM with diabetic chronic kidney disease	106
E11.29	T2DM with other diabetic kidney complication	1
E11.311	T2DM with unspecified diabetic retinopathy with macular oedema	1

Code	Description	Case admissions, n
E11.319	T2DM with unspecified diabetic retinopathy without macular oedema	28
E11.3212	T2DM with mild nonproliferative diabetic retinopathy with macular oedema, left eye	1
E11.3299	T2DM with mild nonproliferative diabetic retinopathy without macular oedema, unspecified eye	3
E11.3319	T2DM with moderate nonproliferative diabetic retinopathy with macular oedema, unspecified eye	1
E11.3392	T2DM with moderate nonproliferative diabetic retinopathy without macular oedema, left eye	1
E11.349	T2DM with severe nonproliferative diabetic retinopathy without macular oedema	1
E11.3519	T2DM with proliferative diabetic retinopathy with macular oedema, unspecified eye	1
E11.3599	T2DM with proliferative diabetic retinopathy without macular oedema, unspecified eye	3
E11.36	T2DM with diabetic cataract	4
E11.40	T2DM with diabetic neuropathy, unspecified	7
E11.42	T2DM with diabetic polyneuropathy	24
E11.51	T2DM with diabetic peripheral angiopathy without gangrene	20
E11.52	T2DM with diabetic peripheral angiopathy with gangrene	2
E11.59	T2DM with other circulatory complications	4
E11.610	T2DM with diabetic neuropathic arthropathy	1
E11.621	T2DM with foot ulcer	6
E11.622	T2DM with other skin ulcer	3
E11.641	T2DM with hypoglycaemia with coma	1
E11.649	T2DM with hypoglycaemia without coma	12
E11.65	T2DM with hyperglycaemia	110
E11.69	T2DM with other specified complication	1
E11.8	T2DM with unspecified complications	2
E11.9	T2DM without complications	531

T2DM: type 2 diabetes mellitus. An admission may contribute more than one code. Descriptions follow the official ICD-10-ES 2024 reference table (Spanish Ministry of Health).

Population-level estimation

Both samples were selected on the result of the index test (test-result-based sampling; Kohn MA, J Clin Epidemiol 2022;145:179–82), so each is a probability sample of its own stratum. The predictive values are therefore estimated directly, whereas sensitivity and specificity cannot be computed from the pooled 2×2 table, because coded admissions were sampled at about four times the fraction of non-coded admissions (787 of 918,240 versus 781 of 3,783,969). Table S2 shows how the pooled estimates depend on the ratio of control to case admissions.

Table S2. Dependence of the pooled sample estimates on the ratio of control to case admissions.

Control admissions per case admission	n (non-coded)	Pooled sensitivity %	Pooled specificity %	PPV % / NPV %
1:1 (design of this study)	781	93.63	95.05	95.17 / 93.47
1:2	1,574	87.93	97.48	95.17 / 93.47
1:4.12 (population ratio)	3,243	77.96	98.76	95.17 / 93.47
1:10	7,870	59.31	99.49	95.17 / 93.47

Proportions within each arm are held at their observed values (95.17% of coded and 6.53% of non-coded admissions met the reference standard); only the number of control admissions varies.

The population classification table was reconstructed within each hospital complexity group h, with N^+_h coded and N^-_h non-coded discharges in the source population: $TP_h = N^+_h \cdot \hat{p}(D+|T+, h)$; $FP_h = N^+_h \cdot [1 - \hat{p}(D+|T+, h)]$; $FN_h = N^-_h \cdot \hat{p}(D+|T-, h)$; $TN_h = N^-_h \cdot [1 - \hat{p}(D+|T-, h)]$ (Wang W et al., DEVELOP-RCD, Mil Med Res 2024;11:52). Sensitivity, specificity, predictive values and prevalence were computed from the summed table, with 95% CIs by parametric bootstrap (200,000 replicates, percentile method).

Post-stratification of the non-coded arm. The matched control sample reproduces the age and sex structure of coded admissions, whereas non-coded discharges are younger (46.5% under 60 years, versus 10.6% of the matched sample) and more often women (56.3%, versus 43.8%). The proportion of reference-positive admissions observed in each age-band–sex cell of the control sample (27 to 147 controls per cell; observed proportions between 0% and 9.6%) was therefore weighted to the corresponding cell of non-coded discharges, with the joint age–sex distribution obtained from the population margins by iterative proportional fitting. The post-stratified proportion was 6.0%, against 7.4% under the matched profile, and was applied to the group-specific denominators, as it did not differ across complexity groups ($p = 0.30$). Table S3 shows the estimates under alternative weighting schemes.

Table S3. Sensitivity of the population-level estimates to the weighting of the non-coded arm.

Proportion of reference-positive admissions among non-coded discharges	Sensitivity	NPV	Prevalence of T2DM
7.4% (matched profile, weighted by hospital group)	75.6	92.6	24.3
6.6% (post-stratified by age only)	77.5	93.4	23.7
6.1% (post-stratified by sex only)	78.9	93.9	23.3
6.0% (post-stratified by age and sex; primary)	79.3	94.0	23.2
5.8% (age and sex, joint assuming independence)	79.6	94.2	23.1

Values are percentages; the fourth row is the primary estimate.

Table S4. Diagnostic validity of type 2 diabetes mellitus (T2DM) coding in the CMBD by hospital complexity group: population-level estimates and pooled sample proportions.

	Population weight, % (Se / Sp)	Sensitivity, % (95% CI)	Specificity, % (95% CI)	PPV, % (95% CI)	NPV, % (95% CI)	Prevalence of T2DM, % (95% CI)
Group 1	44.6 / 49.5	70.75 (61.72–81.30)	97.85 (96.87–98.73)	90.8 (85.9–94.1)	91.8 (87.1–94.9)	23.02 (19.93–26.42)
Group 2	26.3 / 23.5	78.75 (70.65–87.95)	98.99 (98.21–99.70)	96.5 (93.0–98.3)	92.9 (88.5–95.7)	26.17 (23.47–29.17)
Group 3	20.5 / 18.9	78.41 (70.34–87.79)	99.44 (98.85–99.86)	98.0 (95.0–99.2)	92.9 (88.4–95.7)	26.05 (23.34–29.07)
Group 4	8.6 / 8.1	87.00 (79.06–95.84)	98.76 (97.95–99.45)	95.3 (91.3–97.5)	96.3 (92.6–98.2)	22.42 (20.42–24.81)
Validation sample (pooled) [a]	—	93.63 (91.71–95.12)	95.05 (93.28–96.37)	95.17 (93.44–96.46)	93.47 (91.52–95.00)	—
Population level (overall)	—	79.28 (72.39–86.17)	98.51 (97.99–98.99)	94.15 (92.06–96.05)	94.03 (91.29–96.33)	—

CI: confidence interval; PPV: positive predictive value; NPV: negative predictive value; Se: sensitivity; Sp: specificity. Population-level estimates were obtained by reconstructing the classification table within each hospital complexity group, with 409,601, 241,589, 188,533 and 78,517 coded and 1,873,275, 889,538, 715,992 and 305,164 non-coded discharges in Groups 1 to 4. 95% CIs by parametric bootstrap (200,000 replicates), except for the group-specific predictive values (Wilson score). The overall estimates incorporate the age and sex post-stratification of the non-coded arm; the group-specific estimates do not, and are slightly conservative for sensitivity and prevalence. [a] Sample proportions under the 1:1 design (Wilson score CIs); not population estimates.

Figure S3. Positive predictive value of ICD-10-ES code E11 as a function of the prevalence of T2DM in the target population, by hospital complexity group.

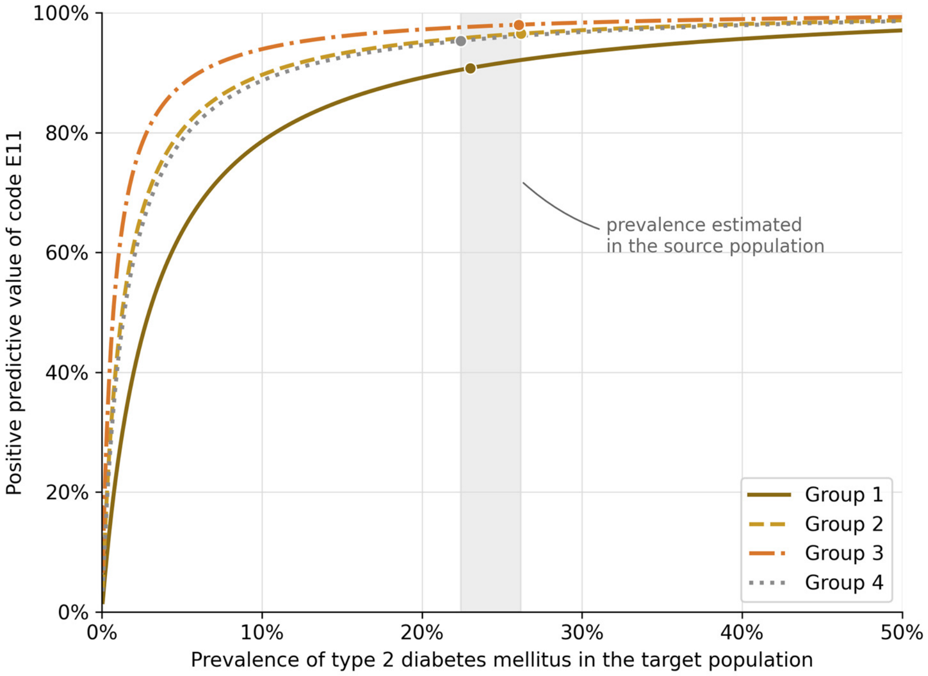

Curves are derived from the population-level sensitivity and specificity of each hospital complexity group; markers indicate the estimated prevalence of T2DM in each group, and the shaded band the range of the group estimates (22.4–26.2%).

Robustness of the results

The 38 coded admissions that did not meet the ADA criteria on the laboratory results available in the EHR up to the index discharge (false positives) were reviewed case by case and grouped into five categories (Table S5).

Table S5. Categories of the 38 false-positive admissions.

Category	n	Rationale for classification as false positive
1. Documented prediabetes or impaired fasting glucose	9	Prediabetes or impaired fasting glucose was documented in the record, and no laboratory value reached the diagnostic thresholds for diabetes.
2. Hyperglycaemia in the context of corticosteroid therapy or acute illness	12	Values in the diabetic range occurred only during high-dose corticosteroid treatment or acute intercurrent illness, without fulfilment of the criteria outside these episodes.
3. Latent autoimmune diabetes of adults or type 1 diabetes	4	Autoimmune diabetes was documented; these admissions do not correspond to T2DM.
4. Glucose-lowering treatment without qualifying laboratory values	7	Glucose-lowering treatment was documented (mostly metformin, in some cases prescribed for prediabetes or other indications), but no qualifying laboratory value was verifiable in the record.
5. No laboratory values in the diabetic range	6	Glucose measurements were available, but none reached the diagnostic thresholds, and no clinical documentation supported a diagnosis of diabetes.

Misclassification in this review can only operate in one direction: if the admissions in category 4, with documented glucose-lowering treatment but no verifiable diagnosis, were true T2DM, specificity and positive predictive value would be underestimated. As a sensitivity analysis, these seven admissions were reclassified as true positives (Table S6).

Table S6. Positive predictive value under the observed classification and after reclassifying the seven category-4 admissions as true positives.

	Observed classification			Category 4 reclassified as TP		
	**TP**	**FP**	**PPV, % (95% CI)**	**TP**	**FP**	**PPV, % (95% CI)**
Group 1	177	18	90.77 (85.88–94.08)	180	15	92.31 (87.70–95.28)
Group 2	193	7	96.50 (92.95–98.29)	193	7	96.50 (92.95–98.29)
Group 3	196	4	98.00 (94.97–99.22)	197	3	98.50 (95.68–99.49)
Group 4	183	9	95.31 (91.33–97.51)	186	6	96.88 (93.35–98.56)
Validation sample (pooled) [a]	749	38	95.17 (93.44–96.46)	756	31	96.06 (94.46–97.21)
Population level [b]	—	—	94.15	—	—	95.07

TP: true positive; FP: false positive; PPV: positive predictive value. Wilson score confidence intervals. [a] Sample proportion under the 1:1 design. [b] Weighted by the share of coded discharges in each hospital complexity group (point estimates). Reclassification does not involve the non-coded arm; the population-level sensitivity would rise from 79.3% to 79.4%.

The proportion of coded admissions meeting the reference standard and the proportion of non-coded admissions not meeting it did not vary across years of discharge or between 2016–2020 and 2021–2025 (Table S7).

Table S7. Classification of the validation sample by year of hospital discharge and by study period.

**Year**	**n**	**TP**	**FP**	**FN**	**TN**	**Coded admissions meeting the reference standard, % (95% CI)**	**Non-coded admissions not meeting it, % (95% CI)**
2016	146	66	3	7	70	95.65 (87.98–98.51)	90.91 (82.40–95.53)
2017	151	77	2	2	70	97.47 (91.23–99.30)	97.22 (90.43–99.23)
2018	144	69	4	8	63	94.52 (86.74–97.85)	88.73 (79.31–94.18)
2019	162	74	5	7	76	93.67 (86.03–97.27)	91.57 (83.60–95.85)
2020	149	72	4	6	67	94.74 (87.23–97.93)	91.78 (83.21–96.18)
2021	159	82	2	5	70	97.62 (91.73–99.34)	93.33 (85.32–97.12)
2022	175	81	7	7	80	92.05 (84.48–96.09)	91.95 (84.31–96.05)
2023	169	79	3	4	83	96.34 (89.79–98.75)	95.40 (88.77–98.20)
2024	170	81	4	2	83	95.29 (88.52–98.15)	97.65 (91.82–99.35)
2025	143	68	4	3	68	94.44 (86.57–97.82)	95.77 (88.30–98.55)
2016–2020	752	358	18	30	346	95.21 (92.56–96.95)	92.02 (88.84–94.35)
2021–2025	816	391	20	21	384	95.13 (92.60–96.83)	94.81 (92.20–96.58)

TP: true positive; FP: false positive; FN: false negative; TN: true negative. Wilson score confidence intervals. The two proportions estimate the positive and negative predictive value in each year or period. χ^2 test between periods: p = 0.96 (coded arm) and p = 0.11 (non-coded arm); across years: p = 0.86 and p = 0.36. Reconstructed to the discharges of each period without post-stratification, the positive predictive value was 94.3% in 2016–2020 and 94.0% in 2021–2025, and the sensitivity 70.5% and 80.6% (difference 10.1 percentage points, 95% CI: −1.0 to 20.7).

Distribution of the continuous variables

Age showed moderate left skewness and length of stay a marked right skewness, and the Shapiro–Wilk test rejected normality for both (Table S8); both are summarised as median and interquartile range in addition to mean and standard deviation.

Table S8. Distribution and normality of the continuous variables (n = 1,568 analysed admissions).

**Variable**	**Mean (SD)**	**Median (IQR)**	**Skewness**	**Shapiro–Wilk W**	**p-value**
Age, years	76.2 (12.1)	77.7 (69.0–85.7)	−0.69	0.967	<0.001
Length of stay, days	10.0 (57.1)	5 (2–10)	36.0	0.063	<0.001

SD: standard deviation; IQR: interquartile range.

Figure S4. Distribution of age in the analysed admissions (n = 1,568).

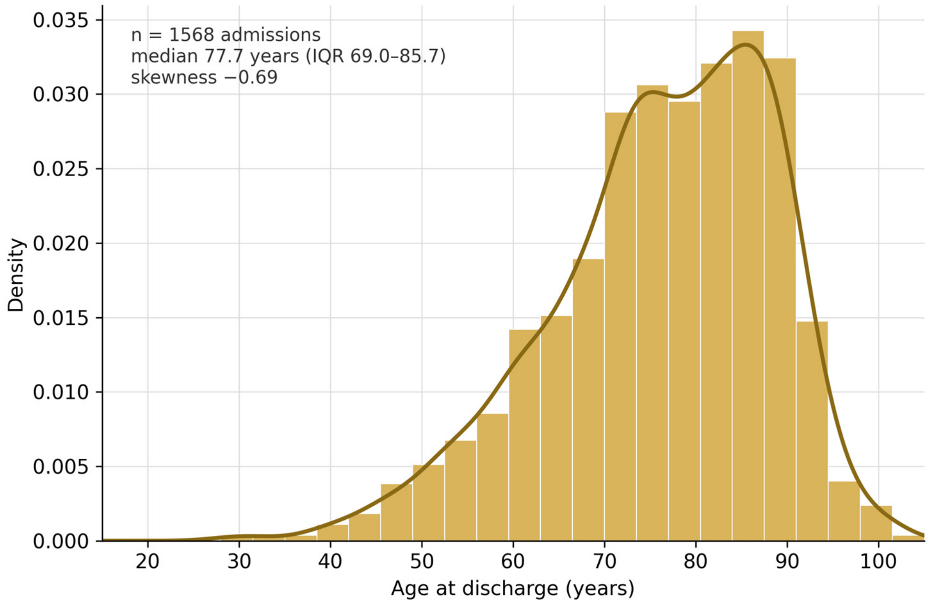

Figure S5. Distribution of length of stay in the analysed admissions (n = 1,568). Fifteen admissions exceeded 60 days and are pooled in the final bin (maximum 2,196 days).

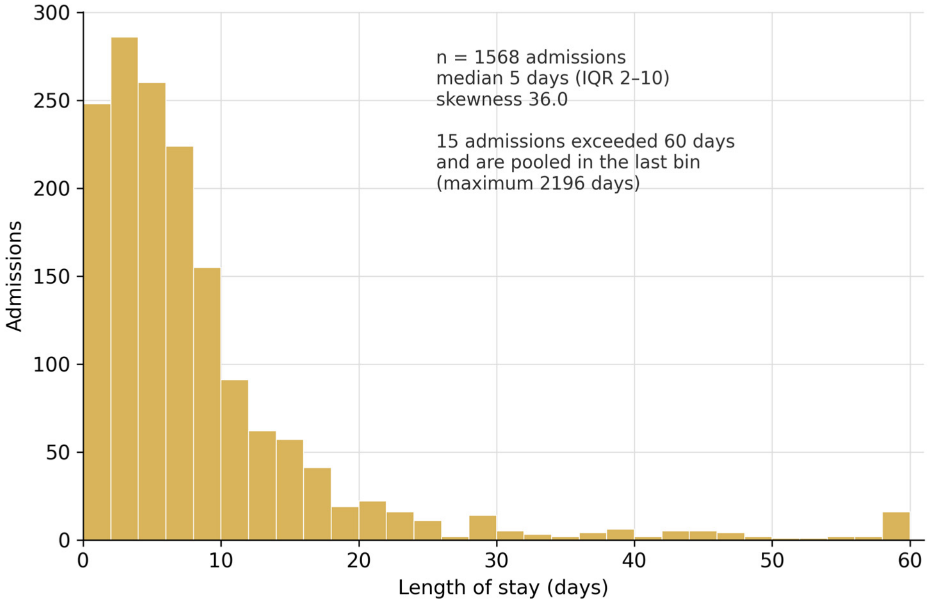

STARD 2015 checklist

Section and item numbering follow Bossuyt PM, et al. STARD 2015: an updated list of essential items for reporting diagnostic accuracy studies. BMJ 2015;351:h5527. "Reported at" refers to the sections, tables and figures of the manuscript and of this document.

Table S9. STARD 2015 checklist.

Section & topic	No	Item	Reported at
**TITLE OR ABSTRACT**	1	Identification as a study of diagnostic accuracy using at least one measure of accuracy (such as sensitivity, specificity, predictive values, or AUC)	Title; Abstract (sensitivity, specificity, PPV, NPV); keyword “validation study”
**ABSTRACT**	2	Structured summary of study design, methods, results, and conclusions (for specific guidance, see STARD for Abstracts)	Abstract (Background/Objectives, Methods, Results, Conclusions)
**INTRODUCTION**	3	Scientific and clinical background, including the intended use and clinical role of the index test	Introduction, paragraphs 1–5 (secondary use of the CMBD; cohort identification and burden estimation)
	4	Study objectives and hypotheses	Introduction, final paragraph
**METHODS – Study design**	5	Whether data collection was planned before the index test and reference standard were performed (prospective study) or after (retrospective study)	Methods, Section 2.1 (retrospective, cross-sectional)
**Participants**	6	Eligibility criteria	Methods, Section 2.3 (adult admissions 2016–2025; exclusion criteria)
	7	On what basis potentially eligible participants were identified (such as symptoms, results from previous tests, inclusion in registry)	Methods, Section 2.5 (presence or absence of ICD-10-ES code E11 in the CMBD)
	8	Where and when potentially eligible participants were identified (setting, location and dates)	Methods, Section 2.2 (SERMAS public hospital network, Region of Madrid, discharges 2016–2025)
	9	Whether participants formed a consecutive, random or convenience series	Methods, Section 2.5; Supplementary Materials, Selection of the validation samples (simple random sampling of coded admissions within each hospital complexity group; controls matched 1:1 on sex and age in years)
**Test methods**	10a	Index test, in sufficient detail to allow replication	Methods, Sections 2.1 and 2.5; Supplementary Table S1 (code E11, all subcodes, any of the 20 diagnostic positions)
	10b	Reference standard, in sufficient detail to allow replication	Methods, Section 2.6 (ADA 2025 diagnostic criteria verified in the EHR across care settings)
	11	Rationale for choosing the reference standard (if alternatives exist)	Methods, Sections 2.1 and 2.6 (ADA criteria are the accepted clinical diagnostic standard; unchanged since 2016)
	12a	Definition of and rationale for test positivity cut-offs or result categories of the index test, distinguishing pre-specified from exploratory	Methods, Section 2.5 (E11 in any diagnostic position; pre-specified)
	12b	Definition of and rationale for test positivity cut-offs or result categories of the reference standard, distinguishing pre-specified from exploratory	Methods, Section 2.6 (ADA thresholds; two abnormal results required in the absence of unequivocal hyperglycaemia; pre-specified)
	13a	Whether clinical information and reference standard results were available to the performers or readers of the index test	Methods, Sections 2.1 and 2.4 (the index test is routine clinical coding, performed before and independently of the study; reference standard results did not exist at the time of coding)
	13b	Whether clinical information and index test results were available to the assessors of the reference standard	Methods, Section 2.6 (both reviewers blinded to coding status and sample assignment; discrepancies resolved by a third reviewer)

Section & topic	No	Item	Reported at
**Analysis**	14	Methods for estimating or comparing measures of diagnostic accuracy	Methods, Section 2.7; Supplementary Materials, Population-level estimation (direct estimation of predictive values; reconstruction of the population classification table; age–sex post-stratification of the non-coded arm; parametric bootstrap)
	15	How indeterminate index test or reference standard results were handled	Methods, Section 2.6; Discussion; Supplementary Tables S5 and S6 (admissions whose criteria could not be verified were classified reference-negative; reclassification sensitivity analysis)
	16	How missing data on the index test and reference standard were handled	Methods, Sections 2.3 and 2.5; Results 3.1; Figure 1 (32 admissions without CIPA excluded; no missing laboratory data in the analysed sample)
	17	Any analyses of variability in diagnostic accuracy, distinguishing pre-specified from exploratory	Methods, Section 2.7; Tables 2–3; Supplementary Tables S4 and S7 (by sex, age group and hospital complexity group, pre-specified; by year and study period, added at revision)
	18	Intended sample size and how it was determined	Methods, Section 2.5 (200 admissions per stratum and sample; calculation reported)
**RESULTS – Participants**	19	Flow of participants, using a diagram	Results 3.1; Figure 1
	20	Baseline demographic and clinical characteristics of participants	Results 3.1; Table 1
	21a	Distribution of severity of disease in those with the target condition	Table 1 (comorbidity burden); Supplementary Table S1 (E11 subcodes, including complicated forms)
	21b	Distribution of alternative diagnoses in those without the target condition	Methods, Section 2.5; Results 3.1 (other diabetes categories eligible as controls; nine control admissions with E09/E10/E13); Supplementary Table S5 (alternative conditions among discordant coded admissions)
	22	Time interval and any clinical interventions between index test and reference standard	Methods, Section 2.4 (EHR reviewed from inception of the record to the index discharge; records-based study, no interventions)
**Test results**	23	Cross tabulation of the index test results (or their distribution) by the results of the reference standard	Results 3.2; Table 2 (classification counts, overall and by stratum)
	24	Estimates of diagnostic accuracy and their precision (such as 95% confidence intervals)	Results 3.2–3.5; Tables 2–4; Supplementary Table S4
	25	Any adverse events from performing the index test or the reference standard	Not applicable (retrospective study of routinely collected records; no tests performed on patients)
**DISCUSSION**	26	Study limitations, including sources of potential bias, statistical uncertainty, and generalisability	Discussion, limitations paragraph
	27	Implications for practice, including the intended use and clinical role of the index test	Discussion (transportability of predictive values, Table 4 and Supplementary Figure S3); Conclusions
**OTHER INFORMATION**	28	Registration number and name of registry	The study was not registered

Section & topic	No	Item	Reported at
	29	Where the full study protocol can be accessed	No published protocol; the sampling and matching specification and the query defining the study universe are reproduced in the Supplementary Materials
	30	Sources of funding and other support; role of funders	Funding statement (no external funding)
